# Doses of exposure to night shift work and nutritional status among nursing workers

**DOI:** 10.47626/1679-4435-2021-618

**Published:** 2021-12-30

**Authors:** Cecília Salazar Ulacia, Aline Silva-Costa, Lúcia Rotenberg, Rosane Härter Griep

**Affiliations:** 1 Instituto de Ciências da Saúde, Universidade Federal do Triângulo Mineiro, Uberaba, MG, Brazil.; 2 Laboratório de Educação em Ambiente e Saúde, Fundação Oswaldo Cruz, RJ, Brazil.

**Keywords:** body weight, waist circumference, night work, occupational health, nutritional status, peso corporal, circunferência abdominal, trabalho noturno, saúde do trabalhador, estado nutricional

## Abstract

**Introduction:**

Exposure to night work (NW) has been identified as a possible risk factor for body weight gain. Recent studies highlight the need to assess the intensity and frequency of exposure to night work (i.e., years of exposure and number of nights worked).

**Objectives:**

To investigate the relationships between the dose of exposure to night work (years working nights and number of nights worked) and nutritional status (excess weight, waist circumference), abdominal obesity, and body mass index in nursing professionals.

**Methods:**

Data were analyzed on night workers (n = 529) from a public hospital in Rio de Janeiro, Brazil. Descriptive analyses were conducted and crude and adjusted regression models were constructed to test the associations between exposures and outcomes. Analyses were performed using R, version 2.15.

**Results:**

Working at night for 10 years or more was associated with excess weight (odds ratio [OR] = 1.76; 95% confidence interval [95%CI] 1.14-2.72), with abdominal obesity (OR = 1.76; 95%CI 1.14-2.74), with increased body mass index (ß = 2.28; 95%CI 1.31-3.26), and with increased waist circumference (ß = 4.63; 95%CI 2.38-6.88), when compared with exposure to NW for less than 10 years, after adjusting for covariates. The current dose of night work only exhibited a borderline association between > 5 nights/fortnight and abdominal obesity (OR = 1.55; 95%CI 1.01-2.01).

**Conclusions:**

Detailing night work exposure can contribute data to support strategies for organizing working hours that consider the possibility of limiting the time exposed to night work.

## Resumo

**Introdução:**

A exposição ao trabalho noturno (TN) vem sendo apontada como possível fator de risco para o aumento do peso corporal. Estudos recentes destacam a necessidade de avaliação da intensidade e da frequência da exposição ao TN (ou seja, anos de exposição e número de noites trabalhadas).

**Objetivos:**

Investigar a relação entre a dose de exposição ao TN (anos e número de noites trabalhadas) e o estado nutricional (excesso de peso, circunferência abdominal, obesidade abdominal e índice de massa corporal) em profissionais de enfermagem.

**Métodos:**

Foram analisados os dados referentes aos trabalhadores noturnos (n = 529) de um hospital público do Rio de Janeiro. Realizaram-se análises descritivas e modelos de regressão brutos e ajustados para testar as associações entre as exposições e os desfechos. As análises foram realizadas no programa R, versão 2.15.

**Resultados:**

Trabalhar à noite por 10 anos ou mais associou-se ao excesso de peso (*odds ratio* [OR] = 1,76; intervalo de confiança de 95% [IC95%] 1,14-2,72), à obesidade abdominal (OR = 1,76; IC95% 1,14-2,74), ao índice de massa corporal aumentado (ß = 2,28; IC95% 1,31-3,26) e à cintura abdominal aumentada (ß = 4,63; IC95% 2,38-6,88), comparado ao TN realizado há menos de 10 anos, após ajuste pelas covariáveis. No que se refere à dose atual de TN, foi observada apenas uma associação limítrofe entre mais de 5 noites/quinzena e a obesidade abdominal (OR = 1,55; IC95% 1,01-2,01).

**Conclusões:**

O detalhamento da exposição ao TN pode contribuir para subsidiar as estratégias de organização dos horários de trabalho que considerem a possibilidade de limitação do tempo de exposição ao TN.

## INTRODUCTION

Night work (NW) is increasingly common in a globalized world. Around 15 to 20% of the populations of Europe and the United States work shifts, including the night shift.^1^ In Brazil, the results of the 2013 National Health Survey showed that 14.9% of working people worked nights.^2^

Workers who work during the night shift have to cope with maladjustment of the circadian rhythm, caused by reversal of sleeping and waking hours, which can trigger many changes to physiological variables.^3^ These changes can cause metabolic deregulation that leads to problems such as increased body mass index (BMI)^4,5^ and waist circumference (WC),^5^ making exposure to NW a possible risk factor for cardiometabolic diseases.^6^

Recent studies have identified that prolonged exposure to NW (number of years working nights) is associated with increased BMI.^7,8^ However, BMI cannot differentiate the cause of excess weight between musculature or adipose tissue,^7^ so it is important to analyze WC, which may be a better predictor of cardiovascular risk.^9^

A recent review article identified a 23% greater risk of overweight/obesity and 35% greater risk of abdominal obesity among night shift workers.^5^ However, the authors emphasized a need for more studies that assess the intensity and frequency of exposure to NW (i.e., years of exposure and number of nights worked) to obtain conclusive results.^5^

Health professionals, and especially nursing workers, constitute one of the professional categories exposed to NW for long periods during their working lives. The objective of this study was therefore to evaluate the association between the dose of exposure to NW (years and nights worked) and nutritional status (BMI, WC, excess weight, and abdominal obesity) among nursing workers.

## METHODS

### STUDY TYPE AND STUDY POPULATION

The population eligible for this cross-sectional study comprised 583 nursing professionals who work providing care at a public hospital in Rio de Janeiro, Brazil. For the purposes of the present study, workers for whom data were missing were excluded, resulting in a sample of 529 nursing workers who worked night shifts.

### DATA COLLECTION

Data were collected at the hospital, during the nursing professionals’ work shifts, in two stages. The first step was administration of a questionnaire covering sociodemographic and lifestyle variables and employment characteristics. The second step comprised anthropometric measurements (weight, height, and waist circumference). The anthropometric assessment followed all of the standardized procedures recommended in the literature.^10^ Both steps were conducted by trained interviewers/examiners.

### DEFINITION OF NW AND VARIABLES ASSESSED

Night workers were defined as nursing professionals who reported working the night shift (from19:00h to 07:00h, at least once per week or four times a month).

#### A. Accumulated NW exposure dose

This variable was assessed using the following question: How long have you been working at night? The results were dichotomized as 1 to 9 years or 10 years or more of NW experience.^11^

#### B. Current NW exposure dose

This variable was assessed as the recall of number of nights worked during the last 2 weeks. The results were dichotomized as two to four nights/fortnight or five or more nights/fortnight.^11^

#### C. BMI

BMI was determined using the participants’ anthropometric measurements, calculated as weight (kg) by height squared (m^2^).

#### D. Excess weight

The excess weight variable was defined as BMI dichotomized as “normal” (< 25 kg/m^2^) or “excess weight” (≥ 25 kg/m^2^).

#### E. WC

WC was defined as waist circumference measured above the umbilical scar, in cm.^12^

#### F. Abdominal obesity

The abdominal obesity variable was defined as WC dichotomized as > 88 cm for women and > 102 for men.^12^

#### G. Covariates

Covariates were assessed as follows. Age: in years; marital status: “married”, “separated/divorced”, “widowed”, or “single”; educational level: “up to secondary education” or “completed higher education”; professional category: “nursing technician” or “nurses”; physical activity: leisure time activities, summed as minutes per week and classified according to the World Health Organization^13^ as: “no physical activity”, “< 150 minutes/week”, or “150 or more minutes/week”. Symptoms of insomnia: assessed according to frequency (“never”, “rarely”, “sometimes”, “almost always”, or “always”) of (i) difficulty going to sleep, (ii) waking early and (iii) interrupted sleep, during the last month. Participants who endorsed always or almost always for at least one of these three complaints were classified as having insomnia.^11^ Duration of sleep: duration of sleep was assessed using the following question: on average, considering all 24 hours of the day, how many hours do you sleep? Include both daytime and nighttime sleep, if appropriate. This variable was dichotomized as duration < 7 h/day and 7h or more /day.

### ANALYSIS OF THE DATA

Descriptive analyses were conducted of the study variables to characterize the study population. Categorical variables were expressed as absolute and relative frequencies and quantitative variables were expressed using means and standard deviations. Crude and adjusted regression models were estimated to test the associations between exposure and outcomes. Odds ratios (OR) and 95% confidence intervals (95%CI) were estimated with logistic regression for binary outcomes (excess weight and abdominal obesity). Linear regression analyses were conducted with gamma family and identity link (ß; 95%CI) for numerical outcomes (BMI and WC). The level of significance was set at 5% and R software, version 2.15, was used for analyses.

### ETHICAL PROCEDURES

The study was approved by the Human Research Ethics Committee, decision number 635/11.

## RESULTS

The population of the present study comprised night workers; 81.1% were women, 33.3% were nurses, 51.2% had 10 or more years’ experience of NW, and 26.1% worked five or more nights/fortnight. Higher frequencies of excess weight and abdominal obesity were observed among workers who had 10 or more years’ experience of NW than among those with fewer than 10 years of NW. There was no statistically significant association with current NW dose (Table 1).

**Table 1:** Sociodemographic and behavioral profile of nocturnal healthcare workers by excess weight and abdominal obesity, Rio de Janeiro, Brazil, 2013

	Excess weight		p-value	Abdominal obesity		p-value
	No n (%)	Yes n (%)		No n (%)	Yes n (%)	
Sex						
Male	29 (29.0)	71 (71.0)	0.351	67 (69.1)	30 (30.9)	< 0.001
Female	148 (34.5)	281 (65.5)		155 (37.3)	261 (62.7)	
Educational level						
Secondary education	45 (27.3)	120 (72.7)	0.054	63 (39.4)	97 (60.6)	0.270
Higher education	132 (36.3)	232 (63.7)		159 (45.0)	194 (55.0)	
Marital status						
Single	60 (43.5)	78 (56.5)	0.011	68 (50.0)	68 (50.0)	0.100
Married	96 (30.9)	215 (69.1)		126 (42.3)	172 (57.7)	
Separated/widowed	21 (26.3)	59 (73.7)		28 (35.4)	51 (64.6)	
Professional category						
Nurse	63 (35.8)	113 (64.2)	0.480	72 (42.6)	97 (57.4)	0.904
Nursing technician	114 (32.3)	239 (67.7)		150 (43.6)	194 (56.4)	
Sleep duration, hours/day						
< 7	90 (32.0)	191 (68.0)	0.516	123 (44.6)	153 (55.4)	0.584
≥ 7	87 (35.1)	161 (64.9)		99 (41.8)	138 (58.2)	
Symptoms of insomnia						
No	136 (36.0)	242 (64.0)	0.066	163 (44.8)	201 (55.2)	0.328
Yes	41 (27.2)	110 (72.8)		59 (39.6)	90 (60.4)	
Physical activity, minutes						
No physical activity	102 (31.0)	227 (69.0)	0.293	121 (37.9)	198 (62.1)	0.001
< 150	26 (36.1)	46 (63.9)		30 (43.5)	39 (56.5)	
≥ 150	49 (38.3)	79 (61.7)		71 (56.8)	54 (43.2)	
Accumulated NW dose, years						
< 10	111 (43.0)	147 (57.0)	< 0.001	129 (52.4)	117 (47.6)	< 0.001
≥ 10	66 (24.4)	205 (75.6)		93 (34.8)	174 (65.2)	
Current NW dose, nights/fortnight						
< 5	138 (35.3)	253 (64.7)	0.161	167 (44.4)	209 (55.6)	0.446
≥ 5	39 (28.3)	99 (71.7)		55 (40.1)	82 (59.9)	
	Mean (SD)			Mean (SD)		
BMI (kg/m^2^)	22.8 (1.6)	30.5 (4.4)	< 0.001	24.1 (2.6)	30.9 (4.7)	< 0.001
WC (cm)	82.6 (6.6)	99.3 (9.9)	< 0.001	84.2 (7.2)	101.1 (9.3)	< 0.001
Age (years)	39.3 (10.1)	43.7 (10.7)	< 0.001	39.8 (10.9)	44.3 (10.7)	< 0.001

BMI = body mass index; NW = night work; SD = standard deviation; WC = waist circumference.

Statistically significant associations were observed between accumulated NW dose and outcomes, after adjustment for covariates. Exposure to NW for 10 years or more was associated with increased WC, by a mean of 4.63 cm, compared to workers who had worked nights for less than 10 years. Additionally, working at night for 10 years or more was also associated with a 76% greater likelihood of abdominal obesity. With regard to BMI, it was observed that working at night for 10 years or more was associated with increased BMI, by a mean of 2.4 kg/m^2^, and an 88% greater chance of excess weight compared with workers who had worked at night for fewer than 10 years. With regard to current NW dose, only a borderline association was observed between five or more nights/fortnight and abdominal obesity (Table 2).

**Table 2:** Association between night work dose and body weight in nursing workers, Rio de Janeiro, Brazil, 2013

	BMI (kg/m^2^)		WC (cm)	
	β	95%CI	β	95%CI
Accumulated dose (≥ 10 years)				
Crude model	2.85	2.01-3.70	6.68	4.71-8.66
Adjusted model	2.28	1.31-3.26	4.63	2.38-6.88
Current dose (≥ 5/fortnight)				
Crude model	0.53	-0.47-1.56	1.71	-0.62-4.08
Adjusted model	0.80	-0.18-1.81	1.93	-0.32-4.21
	**Excess weight**		**Abdominal obesity**	
	**OR**	**95%CI**	**OR**	**95%CI**
Accumulated dose (≥ 10 years)				
Crude model	2.34	1.62-3.41	2.06	1.45-2.95
Adjusted model	1.76	1.14-2.72	1.76	1.14-2.74
Current dose (≥ 5/fortnight)				
Crude model	1.35	1.49-2.26	1.19	0.80-1.78
Adjusted model	1.47	0.96-2.31	1.55	1.01-2.01

Reference categories - accumulated dose: < 10 years; current dose: < 5 nights/fortnight. Model adjusted for age, sex, educational level, marital status, and physical activity level. β coefficients are from linear regression models, and odds ratios (OR) are from logistic regression models.

95%CI = 95% confidence interval; BMI = body mass index; WC = waist circumference.

## DISCUSSION

In the present study, statistically significant associations between accumulated NW dose and the outcomes studied illustrate the importance of investigating factors associated with NW by detailing overall exposure, rather than merely considering current working hours.

There are still few studies in the literature that have detailed the variable NW exposure, considering the number of years worked throughout life and the current frequency of night shift working.^5^ A meta analysis of 28 studies about NW and the risks of specific types of obesity found ORs 2.3 and 3.5 times greater for overweight/obesity and abdominal obesity, respectively, in night workers compared to daytime workers. Additionally, workers of fixed night shifts exhibited a 29% greater risk than those who worked on rotating shifts, which suggests that there is a cumulative effect of NW, and highlights the need to detail this variable.^5^

With regard to the accumulated NW dose, a retrospective study of the last 5 years with nurses aged 20 to 39 years revealed that those who worked at night exhibited significant changes to WC compared to those who worked during the day.^14^ In one prospective study, the effect of NW on BMI was observed in a cohort of nurses.^15^ Irrespective of the number of nights worked, nocturnal workers exhibited significant increases in BMI over 4 years and significant increases in BMI compared to daytime workers.^15^

In turn, in regard to current NW dose, a Polish study with healthcare workers observed an association between number of nights worked (two-to-seven nights/month or eight or more nights/month) and BMI and WC.^7^ Although the number of nights assessed in our study has a similar cutoff point to that used by these authors,^7^ only a borderline association between ≥ five nights/fortnight and abdominal obesity was observed in our study. This apparent contradiction can be explained by the younger mean age of the group in this study and by the cutoff point used to evaluate BMI, which was > 30 kg/m^2^ in the Polish study^7^ but ≥ 25kg/m^2^ in the present study.

A significant association between number of nights worked in the last year and increased BMI was also observed in a Norwegian cross-sectional study with nurses.^16^ It is important to mention that in our study the number of nights worked was assessed for a 15-day period, whereas the period was 1 year in the Norwegian study. Therefore, it is possible that the absence of significant associations with current dose observed in our study is because this metric is a snapshot. As such, the number of nights worked may not illustrate the frequency with which the worker has been exposed over the course of the working life and because changes such as body mass gain and increases in WC do not occur at a single point in time, but over time. Additionally, the differences in results related to current NW dose emphasize the importance of detailed assessment of the number of nights for a longer period, thereby considering frequency and intensity of NW exposure in conjunction.

There are biological mechanisms underlying the associations observed in the study. Working during the night results in hormonal changes that are correlated to appetite (leptin and ghrelin),^17^ and also in lifestyle changes (dietary and physical exercise habits).^18^ With regard to hormones, there is a reduction in leptin levels (activation of the sympathetic nervous system due to sleep deprivation) and an increase in ghrelin levels,^19^ contributing to increase the appetite and, as a result, the number of meals. Additionally, during NW, gastrointestinal processes related to digestion, to absorption, and to storage are compromised (vagal activity is different during the nocturnal period and secretion of gastric and biliary liquids is minimal at night).^20^ Additionally, the reduction in physical activity appears to be related to tiredness caused by work and also to the difficulties that night workers face in participating in types of sporting activities that happen at regular times.^21^

The present study is subject to certain limitations, such as the impossibility of generalization of the results, since it only recruited nursing workers with experience of NW. Additionally, another potential problem with the study could be the healthy worker effect, by which only those who tolerate NW well continue working these hours. This could have excluded from the sample workers with chronic diseases at more advanced stages, and obesity is an important risk factor. In this case, our results are underestimated It should also be noted that there are no data that enable comparison with daytime workers, which would have contributed to understanding the results by means of analysis of the dose-response effect, starting from a complete absence of risk. On the other hand, detailing the variable exposure to NW by intensity and frequency of exposure is a highlight, since it contributes to understanding certain discrepant results in the literature. Additionally, this study also analyzed WC, which is an outcome that has been explored little in the literature (compared to BMI) on working shifts, despite being an important risk factor for a series of cardiometabolic diseases. Finally, we should emphasize that, although the results observed are not from a recently-collected data, the working patterns among the nursing teams at Brazilian hospitals have not undergone changes over recent years, which supports the inference that the results are still representative of the current model.

## CONCLUSIONS

In summary, the present study analyzing the relationship between NW and nutritional status in nursing workers showed that the accumulated NW dose (10 years or more) is associated with increased BMI, with excess weight, with abdominal obesity, and with increased WC, which constitutes a contribution to the literature on the cumulative effects of NW. Since excessive weight, whether overweight or obesity, is associated with reductions in quality of life, in life expectancy, and in functional capacity and with increased mortality, studies of modifiable factors related to increased weight merit foregrounding. Although more studies are needed, including other populations of night workers, our results widen the debate on the effective strategies needed to improve occupational health, considering the possibility of limiting NW exposure time and prioritizing measures to control weight and promote healthy lifestyle habits in this group.
